# Supplementary material for: Targeting anger for COVID-19 prevention: The motivating role of anger on media use and vaccination intention
Source: PLoS One. 2025 Dec 17;20(12):e0338183. doi: 10.1371/journal.pone.0338183 (PMC12711046; doi:10.1371/journal.pone.0338183)
Supplement: S1 File — (DOCX) [file pone.0338183.s001.docx]

**Supporting information**

**S1. Study 2 GPT Prompt**

You are an AI agent that classifies the target of anger in COVID-19-related tweet.

You will follow this guideline:

1. Identification of Anger: Begin by identifying whether the tweet expresses anger. Only tweets with a clear expression of anger or frustration should be considered for further classification. If no specific target of anger is identifiable or if the tweet does not express anger, classify the tweet as "15. Others (Unspecified)." Avoid overusing "15. Others (Unspecified)" for tweets that can reasonably fit into one of the defined categories.
2. Utilization of the Codebook: Refer to the provided codebook, which outlines 15 categories of potential targets of anger related to COVID-19. Use these categories to guide your classification. Each category is defined by specific criteria, including descriptions and examples. Ensure that the classification aligns with these definitions.

In the training dataset, look at "Text" column for tweets, and "Target_anger" column for classification results that are human-coded.

1. Based on these, you will code each tweet in the testing dataset. fill in the "Target_anger" column. In your classification, indicate the full category name.
2. Provide a json format code output.

**Codebook**

1. COVID-19 virus and its negative social and health effects on people

- Definition: Expressions of anger directed specifically at the COVID-19 virus and the direct social and health-related consequences it causes, including illness, death, and disruption of daily life.
- Examples:
  - "I hate COVID for taking my grandfather from us."
  - "COVID sucks! It's ruined everything, from jobs to health."
- Guidelines: Include tweets expressing general frustration or anger specifically at the virus and its immediate effects on personal and community health.

2. Vaccine and vaccine mandate

- Definition: Anger towards COVID-19 vaccines and vaccine mandates. This includes skepticism about vaccine safety, efficacy, and anger towards policies requiring vaccination for travel, etc.
- Examples:
  - "Mandating the vaccine is wrong! People should have a choice."
  - "I don't trust the COVID vaccine at all. Too rushed."
- Guidelines: Focus on tweets expressing distrust or frustration with vaccines and mandates. Distinguish between personal skepticism and broader policy criticism.

3a. Personal dislike of wearing masks and social distancing

- Definition: Personal anger or frustration with mask-wearing and social distancing measures, including discomfort and inconvenience caused by these precautions.
- Examples:
  - "Wearing masks all day is unbearable."
  - "Social distancing makes me feel so isolated. Hate it."
- Guidelines: Include only tweets expressing personal inconvenience or discomfort. Do not include criticism of others' non-compliance here.

3b. Anger towards those not adhering to mask-wearing and social distancing guidelines

- Definition: Criticism or anger towards individuals or groups who fail to adhere to recommended health guidelines, such as mask-wearing and social distancing.
- Examples:
  - "So angry at people in the store without masks. It's selfish!"
  - "Why can't everyone just follow the distancing guidelines?"
- Guidelines: Focus on tweets that express frustration with perceived negligence or disregard for public health recommendations.

4. President Trump's handling of COVID-19

- Definition: Anger or criticism directed at President Trump’s management of the COVID-19 pandemic, including policy decisions, public statements, and overall leadership during the crisis.
- Examples:
  - "Trump's disregard for science in handling COVID is infuriating."
  - "Can't believe Trump downplayed the virus. So reckless."
  - “@realDonaldTrump We are not angry. We want a vaccine. We just do not trust you. You are politicizing a vaccine to get votes. You have pressured government organizations to falsify information in order to make you look good. You have had the FDA authorize”
- Guidelines: Include tweets that are specifically critical of Trump's actions or policies related to COVID-19. Ensure the criticism is directly related to Trump’s pandemic response, not general political dissatisfaction.

5. President Biden’s handling of COVID-19

- Definition: Anger or criticism directed at President Biden’s management of the COVID-19 pandemic, including policy decisions, public statements, and overall leadership during the crisis.
- Examples:
  - "Biden's vaccine mandates are divisive and wrong."
  - "So frustrated with Biden's handling of the pandemic."
- Guidelines: Include tweets that are specifically critical of Biden's actions or policies related to COVID-19. Ensure the criticism is directly related to Trump’s pandemic response, not general political dissatisfaction.

6. Government, governor, politicians or political parties handling COVID-19

- Definition: Anger directed at various government officials, governors, politicians, and political parties for their handling of COVID-19. This includes policy decisions, communication, and perceived effectiveness.
- Examples:
  - "Our governor's COVID restrictions are killing small businesses."
  - "The government's slow response to the pandemic is unacceptable."
- Guidelines: Broader than categories 4 or 5, this includes any political figure or entity involved in COVID-19 response, not limited to the presidential level.

7. Jealousy or envy experienced when others are given the opportunity to receive a COVID-19 vaccine

- Definition: Expressing jealousy, envy, or anger towards the fact that others are getting the vaccine before me. Frustration of people cutting in the line.
- Examples:
  - "Why did they get the vaccine before me? So unfair."
  - "Feeling left out and frustrated I can't get my vaccine yet."
  - “jealous i want the vaccine so bad”
- Guidelines: Focus on personal feelings of being overlooked or unfairly treated in the vaccine distribution process.

8. Anti-vaxxers or vaccine-hesitant individuals for COVID-19

- Definition: Anger towards individuals or groups that oppose vaccination or exhibit hesitancy towards the COVID-19 vaccine.
- Examples:
  - "Anti-vaxxers are endangering us all with their ignorance."
  - “I can't stress enough how frustrating anti-vaxers are._x000D_”
- Guidelines: Target specifically those expressing opposition to or skepticism of COVID-19 vaccines, not general vaccine discourse.

9. Misinformation spreaders of COVID-19

- Definition: This category specifically targets expressions of anger directed at individuals, groups, or entities that propagate misinformation, disinformation, fake news, or conspiracy theories about COVID-19. This includes but is not limited to false information about vaccines, treatments, transmission, and prevention measures.
- Examples:
  - "Can't believe there are still people spreading lies about the vaccine containing chips. #Frustrated"
  - "Seeing influential figures downplay the virus is beyond infuriating."
  - "The sheer amount of fake news about COVID on my feed is maddening. Stop spreading lies!"
- Guidelines: The focus should be on the act of spreading misinformation. The tweet must explicitly express anger towards the dissemination of false or misleading information about COVID-19. Tweets that merely mention misinformation without expressing anger towards the spreaders should not be classified here. If the tweet also expresses anger towards a specific policy or authority figure but intertwines this with anger at misinformation, consider the primary target of anger for classification.

10. Employers mandating COVID-19 vaccination*

- Definition: Expressions of anger towards employer-enforced COVID-19 vaccination policies, including mandatory vaccination as a condition for returning to work or continuing employment.
- Examples:
  - "It's outrageous that my job is forcing me to get vaccinated or face termination!" "
  - My company mandating the COVID vaccine is a violation of personal freedoms. Extremely angry about this."
- Guidelines: Include tweets that express frustration or anger specifically at employers or workplace policies related to mandatory vaccination. Exclude general opposition to vaccines.

11. Public health officials during COVID-19

- Definition: Criticism and anger directed at public health officials and entities for their handling of the pandemic, including limited vaccine supplies, vaccine distribution inequities (prioritizing other populations), perceived negligence towards marginalized communities, and insufficient promotion of or safety measures for COVID-19 vaccination.
- Examples:
  - "Furious at the CDC for not prioritizing vaccines for essential workers first. Complete oversight."
  - "Public health officials have failed to ensure fair vaccine access to all communities, and I'm livid about it."
- Guidelines: Focus on tweets targeting the actions or policies of public health officials or organizations. Include issues related to vaccine distribution, prioritization, and public health advisories.

12. Pharmaceutical companies handling COVID-19

- Definition: Anger towards pharmaceutical companies over issues such as perceived profiteering from the pandemic, delays in vaccine production, and lack of transparency in vaccine development and distribution.
- Examples:
  - "Big Pharma's greed during the pandemic, especially with vaccine pricing and patents, is infuriating."
  - "Angry at the slow vaccine rollout. Pharma companies should've been better prepared."
- Guidelines: Classify tweets that specifically target pharmaceutical companies' actions or policies related to COVID-19. This can include criticisms of vaccine availability, costs, and ethical considerations.

13. Medical institutions or Healthcare system

- Definition: Expressions of frustration and anger directed at hospitals, pharmacies, and the broader healthcare system over issues like lack of vaccine sites, difficulty booking appointments, appointment cancellations, and local patient care.
- Examples:
  - "Hospital canceled my vaccine appointment last minute. Utterly furious and let down."
  - "The healthcare system's chaotic vaccine management has left me angry and confused. Why is it so hard to get clear info?"
- Guidelines: Include tweets criticizing the operational aspects of healthcare institutions during the pandemic, especially regarding vaccine booking systems and patient care. Exclude criticisms of individual healthcare workers or prioritization of certain populations.

14. World health organization handling COVID-19

- Definition: Anger and dissatisfaction with the World Health Organization's (WHO) pandemic response, including criticism of policies, guidelines, and the organization's effectiveness in managing global health during COVID-19.
- Examples:
  - "The WHO's flip-flopping on mask guidance has been frustrating and damaging to public trust."
  - "Angry at the WHO for not holding countries accountable for underreporting cases. Their oversight is a global failure."
- Guidelines: Focus on tweets that directly criticize the WHO's actions, decisions, or perceived lack of action in relation to the COVID-19 pandemic. This includes policy guidance, communication, and international coordination efforts.

15. Others (unspecified)

- Definition: This category encompasses tweets where anger is expressed in relation to COVID-19 but does not specifically target any of the entities or issues outlined in categories 1-14. This includes general expressions of frustration or anger about the situation without a clear target, or where the target is outside the predefined categories.
- Examples:
  - "I'm so tired of this. Everything about it makes me angry."
  - "Angry and frustrated but don't even know where to direct it anymore with everything going on."
- Guidelines:
  - Use this category for tweets where the source of anger is vague, generalized, or not directed at a specific entity or issue related to COVID-19.
  - Mentioning Johnson, the politician should be coded as Others (unspecified).
  - Focusing on the topic of abortion, pro-choice, women’s choice should be coded as Others (unspecified).
  - Stating facts or opinions about the COVID-19 virus without expression of anger should be coded as Others (unspecified).

*Note*. * This category was deleted due to low occurrence (n = 1).
