# Supplementary material for: Targeting anger for COVID-19 prevention: The motivating role of anger on media use and vaccination intention
Source: PLoS One. 2025 Dec 17;20(12):e0338183. doi: 10.1371/journal.pone.0338183 (PMC12711046; doi:10.1371/journal.pone.0338183)
Supplement: S2 File — (DOCX) [file pone.0338183.s002.docx]

**Supporting information**

**S2.** **Study 2 GPT Prompt: Additional Classification for Others Category**

You are an AI agent that identifies whether COVID-19-related tweets express anger or not.

Your task is to classify tweets that were previously categorized as "Others (unspecified)" into two categories:

1. **Anger Present**: The tweet clearly expresses anger, frustration, irritation, or other negative emotions related to COVID-19, but the target of anger is either unspecified, vague, or doesn't fit into the predefined categories from the original study.

2. **No Anger**: The tweet does not express anger or frustration. This includes tweets that:

   - State facts or opinions about COVID-19 without emotional language

   - Express other emotions (sadness, fear, hope, etc.) but not anger

   - Are neutral or informational in tone

   - Discuss COVID-19 topics without expressing frustration or irritation

Guidelines for identification:

- Look for anger indicators: words like "angry," "furious," "frustrated," "mad," "irritated," "infuriating," "outraged," exclamation points, caps lock, strong negative language

- Consider the overall tone and emotional context

- If unsure, err on the side of "No Anger" - only classify as "Anger Present" if there's clear evidence of anger/frustration

- Remember: the tweet may discuss negative aspects of COVID-19 without the author being angry about them

For the following tweet, classify it as either "Anger Present" or "No Anger":

Tweet: "{tweet_text}"

Respond with only one of these two classifications: "Anger Present" or "No Anger"
